# Tobacco's dual genomic footprints in bladder cancer revealed by multi-omics analysis: An SBS4-like LumU-enriched signature and smoking-driven HRD-related genomic instability

**DOI:** 10.1016/j.gendis.2025.102001

**Published:** 2025-12-23

**Authors:** Xiang-Yu Meng, Ming-Jun Shi, Shuo Li, Min Liu, Jacqueline Fontugne, Jian Song, Francois Radvanyi, Fu-Bing Wang, Isabelle Bernard-Pierrot, Xing-Huan Wang

**Affiliations:** a Hubei Provincial Clinical Medical Research Center for Nephropathy, Hubei Minzu University, Enshi, Hubei 445000, China; b Institut Curie, CNRS, UMR144, Molecular Oncology Team, PSL Research University, Paris 75005, France; c Department of Urology, Zhongnan Hospital of Wuhan University, Wuhan, Hubei 430071, China; d Wuhan Research Center for Infectious Diseases and Cancer, Chinese Academy of Medical Sciences, Wuhan, Hubei 430071, China; e Department of Urology, Beijing Friendship Hospital, Capital Medical University, Beijing 100050, China; f Department of Clinical Laboratory, Institute of Translational Medicine, Renmin Hospital of Wuhan University, Wuhan, Hubei 430060, China; g Department of Clinical Laboratory Medicine, Zhongnan Hospital of Wuhan University, Wuhan, Hubei 430071, China; h Université Paris-Saclay, Université Versailles St-Quentin, Montigny-le-Bretonneux 78180, France

Tobacco smoking is the primary determinant of bladder cancer (BCa) risk,^1^ yet the field has been puzzled by the absence of the canonical tobacco-associated mutational signature, COSMIC SBS4, in BCa tumors. This discrepancy suggests either a different carcinogenic mechanism or limitations in previous analyses that linked smoking to clock-like SBS5 variants or SBS92.^2^ To resolve this, we performed an integrated multi-omics analysis of 410 muscle-invasive bladder cancers (MIBCs) from The Cancer Genome Atlas (TCGA). Our investigation identified a robust SBS4-like signature in BCa and revealed that tobacco's influence extends beyond point mutations to induce profound genomic instability and oncogenic transcriptional reprogramming.

We began by a *de novo* extraction of mutational signatures from whole-exome sequencing data, which consistently yielded a signature with high similarity (cosine similarity = 0.86) to COSMIC SBS4, hereafter termed SBS4-like. We also identified signatures resembling SBS92 and SBS3, termed SBS92-like and SBS3-ms, respectively (Fig. 1A, B; Fig. S1–S3). To validate their link to tobacco, we found that all three signatures displayed a significant mutational bias on the untranscribed DNA strand, a known consequence of nucleotide excision repair acting on bulky DNA adducts (Fig. 1C). Crucially, the mutational burden of both the SBS4-like and SBS92-like signatures was significantly elevated in current and recent smokers compared to never-smokers and long-term reformed smokers, and was associated with CC>AA mutations, a hallmark of tobacco carcinogens (Fig. 1D).^2^ A limitation of this retrospective analysis is the absence of accurate granular “pack-years” data in the TCGA cohort, which precludes a more precise dose–response correlation. The presence of these tobacco-related mutational signatures was validated in an additional cohort of 192 BCa tumors by mutational signature fitting (Fig. S4).^3^

**Figure 1:**
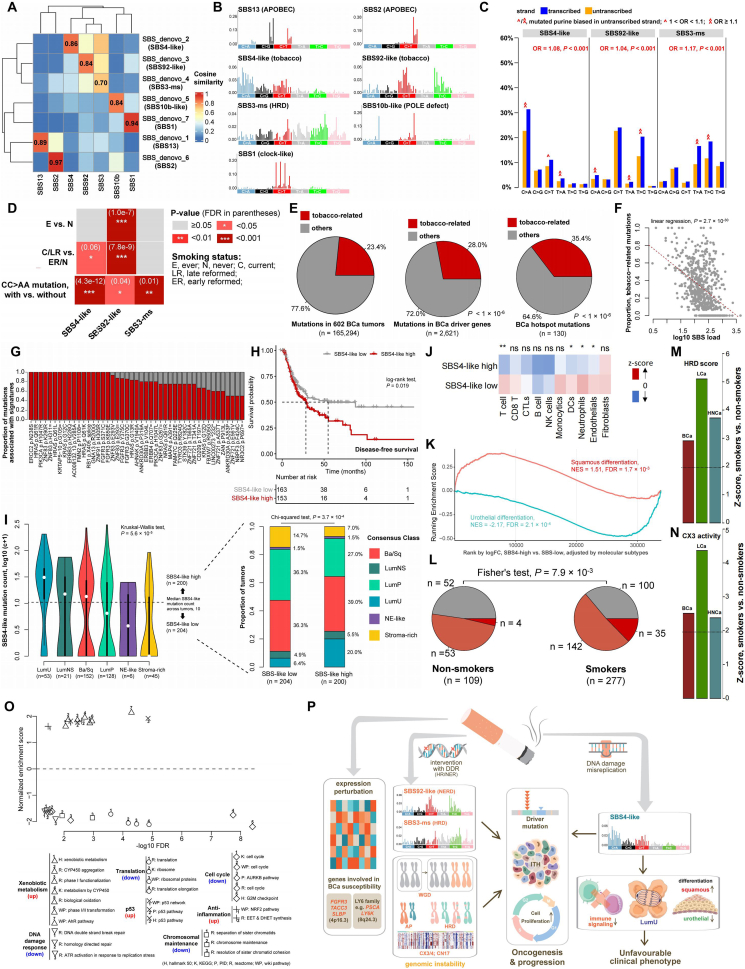
Multifaceted role of tobacco in bladder cancer uncovered by multi-omics analysis. **(A)** Cosine similarity between *de novo* identified and COSMIC reference mutational signatures. The *de novo* extracted mutational signatures were initially named ‘SBS_denovo’ with a sequential suffix number and subsequently renamed per similarity to the reference signatures. Signatures with cosine similarity ≥ 0.9 to a COSMIC signature were given the corresponding name. Signatures with a similarity of ∼0.8 were given a “-like” suffix (*e.g.*, SBS4-like). One signature with moderate similarity to SBS3 was termed SBS3-ms (ms for moderate similarity). **(B)** Substitution profiles of *de novo* extracted mutational signatures and the proposed etiology of the corresponding reference signature shown in brackets. **(C)** Transcriptional strand bias of tobacco-related mutational signatures in BCa. The bars showed the proportion of mutated purine in the untranscribed (yellow) and transcribed (blue) strands. The odds ratio and *P*-value for the mutated purine in the untranscribed strands were calculated for each substitution type. **(D)** Association between smoking history and tobacco-related mutational signatures in BCa, as well as between presence of CC>AA mutation and mutational signatures in BCa. **(E)** Mutational contribution of tobacco-related SBS mutational signatures. Proportion of mutations associated with tobacco-related mutational signatures among overall mutations observed (left). Proportion of mutations related to tobacco-related mutational signatures among the mutations in 45 BCa driver genes (middle) and 130 BCa hotspot mutations (right). *P*-value calculated from sampling permutation test with average background. Data from whole-exome sequencing of 602 BCa tumors (TCGA plus additional 192 tumors, as described previously^3^). **(F)** Relationship between the tumor mutation burden and the proportion of mutations associated with the tobacco-mutational signatures. **(G)** Contribution of tobacco-related mutational signatures in 130 BCa hotspot mutations, shown individually. **(H)** Kaplan–Meier curve of disease-free survival (DFS) in patients with tumors of high (red) and low (grey) SBS4-like mutation load. The dashed lines showed the median DFS. **(I)** Relationship between the SBS4-like mutation load and tumor molecular subtypes defined by the consensus classification framework. **(J)** The relationship between the SBS4-like load and the tumor microenvironment was digitally inferred based on transcriptomic profiles. **(K)** The relationship between the SBS4-like mutation load and the molecularly defined urothelial/squamous differentiation status was determined by molecular subtype-adjusted DEG analysis followed by GSEA. Decreased urothelial differentiation and increased squamous differentiation were observed in SBS4-like high tumors compared to SBS4-like low tumors. **(L)** Relationship between tobacco smoking (never smokers versus ever smokers) and whole-genome duplication (WGD) in TCGA BLCA tumors. **(M, N)** Tobacco-related homologous recombination deficiency (HRD) genomic instability features, the HRD score and the CX3 signature, in BCa and lung and head-and-neck cancers, were analyzed using the Wilcoxon test. A positive Z-score indicates a high level of smokers compared with non-smokers. The dashed line represents the threshold for statistical significance, where an absolute Z-score > 1.96 shows a statistically significant difference. **(O)** Transcriptionally perturbed pathways or gene ontologies upon BaP treatment in RT4 BCa cell line (GSEA analysis of DEG). **(P)** Graphical summary of the multifaceted role of tobacco smoking in bladder cancer.

While APOBEC-related mutagenesis is the dominant mutational force in BCa,^3^ tobacco's impact is more strategic. Tobacco-related signatures accounted for less than a quarter of the total mutation burden (Fig. 1E), and were inversely correlated with it, contrary to the APOBEC-related ones (Fig. 1E, F; Fig. S5, S6). This suggests that tobacco acts as a ‘sniper’, with its mutations being significantly enriched in 45 known BCa driver genes and 130 mutational hotspots (Fig. 1E).^3^ For instance, oncogenic mutations in genes like *KRAS* (G12C) and *HRAS* (Q61 R/L), were predominantly of tobacco-related origin, indicating an outsized oncogenic impact (Fig. 1G; Fig. S7).

Focusing on the canonical SBS4-like signature, we explored its clinical and biological ramifications. No sex bias was observed (Fig. S8), while a high SBS4-like mutation load was an independent predictor of worse disease-free survival (multivariate HR = 1.61, *P* = 7.5 × 10^−3^; Fig. 1H). Molecularly, SBS4-like high tumors were significantly enriched in the Luminal Unstable (LumU) subtype (*P* = 5.3 × 10^−5^), an aggressive classification defined by genomic instability and poor prognosis (Fig. 1I).^4^ Gene set enrichment analysis (GSEA) of their transcriptomes provided a biological explanation, revealing that SBS4-like high tumors had significant up-regulation of cell cycle and proliferation pathways alongside a profound suppression of immune and inflammatory signaling, including interferon and T-cell pathways (Fig. S9). This ‘immune-cold’ phenotype, which we confirmed via digital cytometry showing lower infiltration of T-cells, lower TCR diversity, as well as lower numbers of dendritic cells and neutrophils, provides a molecular rationale for the poorer outcomes observed, which is also a known mechanism of resistance to immune checkpoint inhibitors (Fig. 1J; Fig. S9). Furthermore, these tumors showed a loss of urothelial differentiation and a gain of squamous features, a hallmark of aggressive, therapy-resistant disease, further cementing the link between the SBS4-like signature and a poor prognosis (Fig. 1K). Consistent results were also observed in the NMIBC UROMOL cohort,^1^ where mutational signature fitting identified the SBS4-like signature associated with smoking history, progression risk, and aggressive Class 2a molecular subtype (Fig. S10), further supporting its potential utility in bladder cancer risk stratification and disease management in both MIBC and NMIBC.

The impact of tobacco extends beyond single nucleotide variants to large-scale genomic architecture. We discovered that tumors from smokers had a significantly higher incidence of whole-genome duplication and greater overall aneuploidy than tumors from never-smokers (Fig. 1L; Fig. S11, S12). Most critically, we uncovered a strong association between smoking and homologous recombination deficiency (HRD). Smokers' tumors exhibited significantly higher HRD scores and enriched activity of HRD-associated chromosomal instability (CX3) and copy number (CN17) signatures, which was also observed in lung and head-and-neck cancers (Fig. 1M, N; Fig. S11–S13). This state of HRD, a critical deficiency in the cell's ability to accurately repair DNA double-strand breaks, creates a profound dependency on alternative, error-prone repair pathways. This dependency is the basis for synthetic lethality, where inhibiting a second pathway, in this case, using PARP inhibitors, leads to catastrophic DNA damage and the selective death of the cancer cells. This finding is of potential clinical relevance, as it suggests that BCa in smokers may be more likely vulnerable to this class of targeted therapy.

Finally, to investigate non-mutagenic effects, we explored how tobacco carcinogens directly alter gene expression. We treated the RT4 BCa cell line with benzo[a]pyrene (BaP), a key carcinogen in tobacco smoke. Bulk RNA-sequencing revealed that BaP treatment perturbed critical cellular pathways, activating xenobiotic metabolism and p53 signaling, likely as a stress response, while suppressing G2/M checkpoint control and DNA replication (Fig. 1O).

We further observed a significant overlap between the BaP-responsive genes and bladder cancer susceptibility genes identified by genome-wide association studies (Fig. S14 and Table S1).^5^ For example, dysregulation of *LYNX1* and *PSCA* at the 8q24.3 locus may influence nicotinic acetylcholine receptor signaling, a pathway implicated in nicotine-mediated cell growth.^5^ Considering a list of known oncogenes and tumor suppressor genes (TSGs) already curated in our previous work,^3^ we also observed up-regulation of key oncogenes, such as *MYC*, *CCN1D*, and *KRAS,* and down-regulation of TSGs such as *ATM* and *KMT2A,* after BaP treatment (Table S2). These data suggest that tobacco carcinogens may contribute to urothelial tumorigenesis not only through direct mutagenesis but also by reshaping the transcriptomic landscape.

However, we note that this perturbation experiment models early carcinogenic responses and may not fully capture the transcriptional changes characteristic of late-stage MIBC. We also acknowledge that these observations are hypothesis-generating, and further functional studies will be required to determine whether dysregulation of these genes (susceptibility, oncogenes, and TSGs) is a necessary driver of tobacco-associated bladder carcinogenesis.

In conclusion, our integrative analysis resolves the long-standing paradox of tobacco's mutational footprint in bladder cancer by clearly identifying an SBS4-like mutational signature and distinguishing two tobacco-related mutagenic processes: direct DNA damage (SBS4-like) and altered DNA damage response (SBS92-like and SBS3-ms). As illustrated in Figure 1P, tobacco smoking promotes targeted mutations in key driver genes, induces HRD and genomic instability, favors the LumU molecular subtype and squamous differentiation, and suppresses immune infiltration while perturbing the expression of bladder cancer susceptibility genes and key oncogenes. Importantly, the SBS4-like signature emerges as an independent predictor of worse disease-free survival, while smoking-associated HRD suggests vulnerability to PARP inhibitors, and the corresponding immune-cold phenotype implies potential resistance to immune checkpoint blockade alone. These findings pave the way for the exploration of combination therapeutic strategies, such as PARP inhibition with immunotherapy, for patients with tobacco-associated bladder tumors. Overall, this work provides a mechanistic framework for understanding tobacco-induced bladder carcinogenesis with direct implications for prognosis, patient stratification, and targeted therapy.

## CRediT authorship contribution statement

**Xiang-Yu Meng:** Writing – review & editing, Writing – original draft, Software, Methodology, Investigation, Formal analysis, Conceptualization. **Ming-Jun Shi:** Writing – original draft, Formal analysis, Data curation. **Shuo Li:** Writing – original draft, Formal analysis, Data curation. **Min Liu:** Writing – review & editing, Investigation. **Jacqueline Fontugne:** Writing – review & editing, Investigation. **Jian Song:** Resources, Investigation. **Francois Radvanyi:** Writing – review & editing, Resources. **Fu-Bing Wang:** Writing – review & editing, Supervision, Conceptualization. **Isabelle Bernard-Pierrot:** Writing – review & editing, Supervision, Conceptualization. **Xing-Huan Wang:** Writing – review & editing, Supervision, Conceptualization.

## Funding

This work was supported by la ligue contre le cancer (Equipe labelisée, XYM, JF, FR, IBP), the CAMS Innovation Fund for Medical Sciences (No. 2022-I2M-C&T-B-118), and the SEnACLE project (No. 2019-1-TABAC-02-1). This work was also funded by the Translational Medicine and Interdisciplinary Research Joint Fund and the medical Sci-Tech innovation platform of Zhongnan Hospital of Wuhan University (China) (No. ZNJC202210, PTXM2021001). MJS was supported by the National Natural Science Foundation of China (No. 82002672, 82373436) and Beijing Hospitals Authority's Youth Program (China) (No. QML20230114). XYM was supported by a fellowship from ITMO Cancer AVIESAN within the framework of Cancer Plan, the National Natural Science Foundation of China (No. 82303057), the Natural Science Foundation of Hubei Province of China (No. 2023AFB521), and the “Chutian Scholars Program” of Hubei Province of China. SL was supported by the Hubei provincial critical laboratory open project: seed fund of Renmin Hospital of Wuhan University (No. 2023KFZZ004).

## Conflict of interests

The authors declare that they have no competing interests.
