# Supplementary material for: Tobacco's dual genomic footprints in bladder cancer revealed by multi-omics analysis: An SBS4-like LumU-enriched signature and smoking-driven HRD-related genomic instability
Source: Genes Dis. 2025 Dec 23;13(6):102001. doi: 10.1016/j.gendis.2025.102001 (PMC13380158; doi:10.1016/j.gendis.2025.102001)
Supplement: Multimedia component 1 [file mmc1.docx]

SUPPLEMENTRAY FIGURES AND TABLES


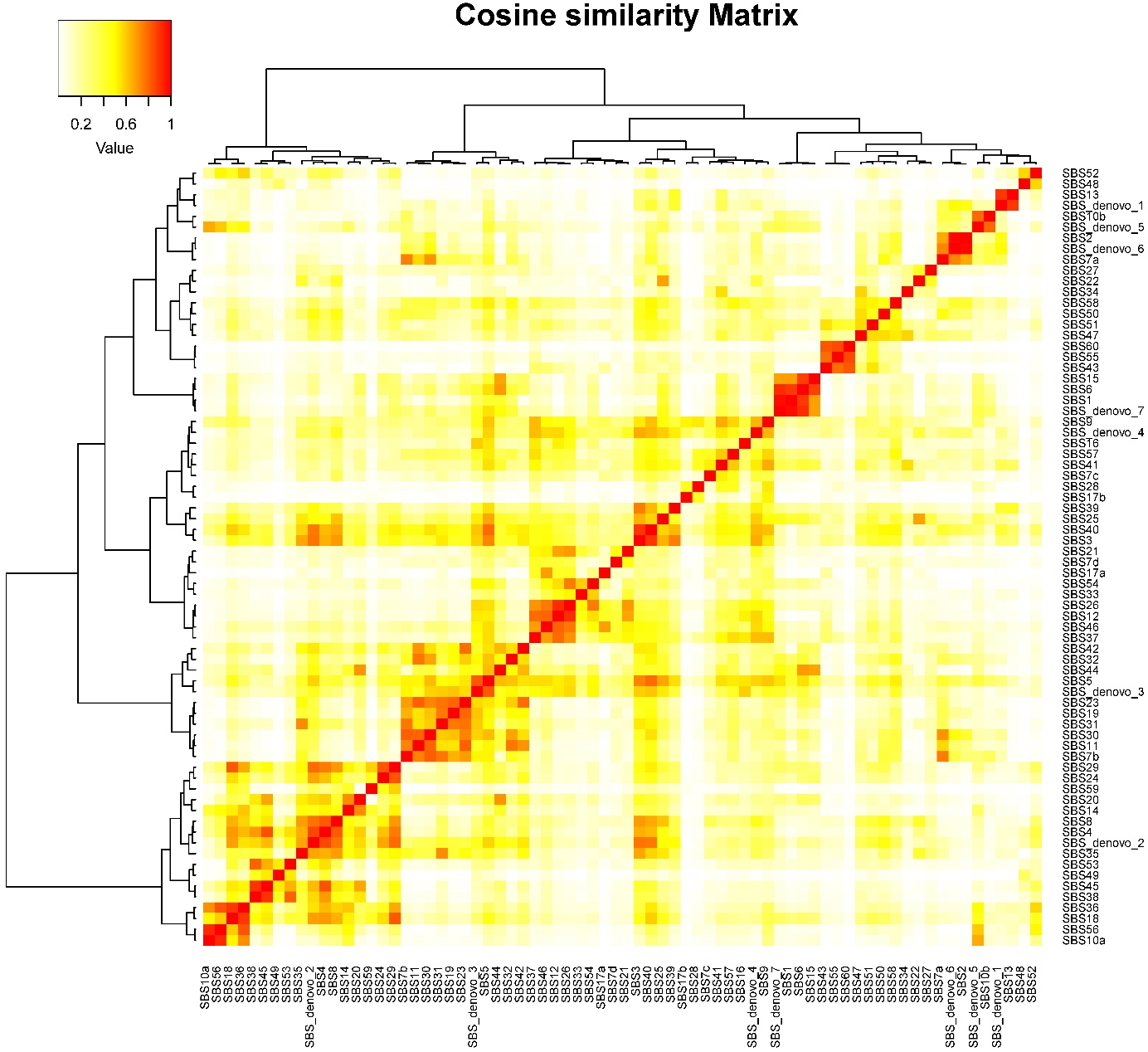


**Figure S1.** **Cosine similarity matrix among the *de novo* extracted mutational signatures (n = 7) in BCa and catalog of signatures in the COSMIC mutational signature database (n = 65).**


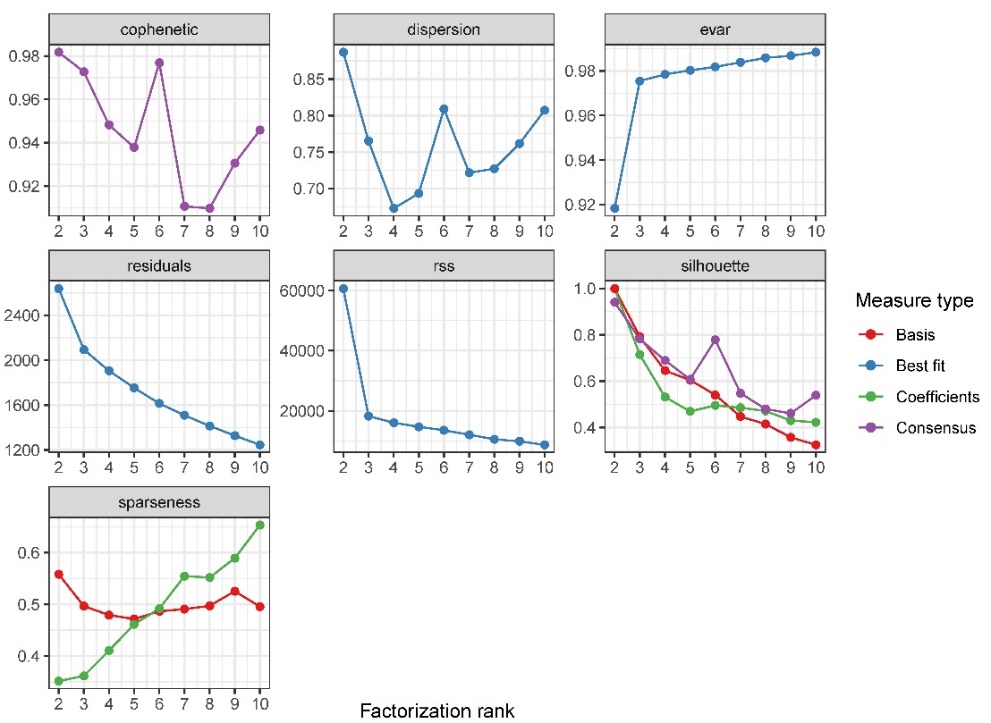


**Figure S2.** **NMF rank survey plots for determining the optimal rank k. Given the metrics, k = 7 was determined to be** optimal for de novo extraction of mutation signatures from TCGA WES data.


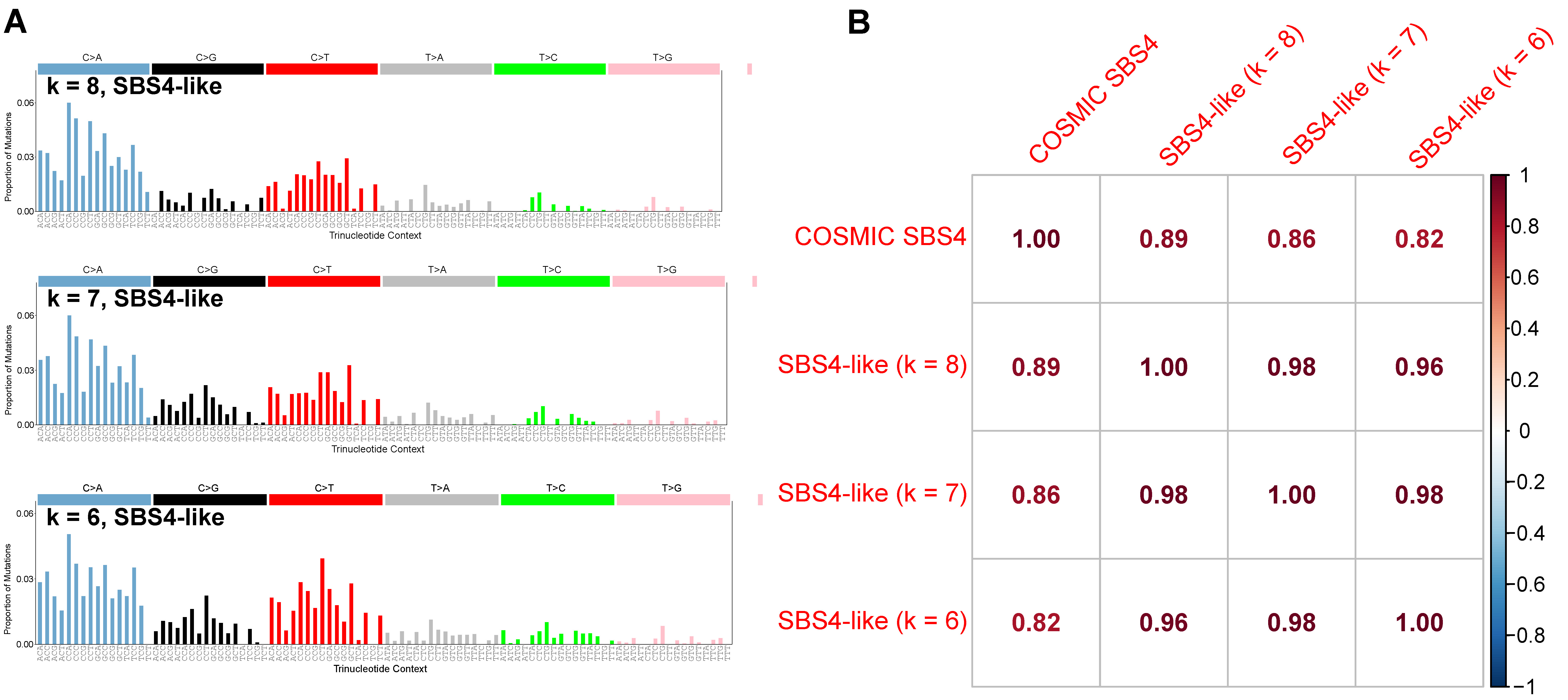


**Figure S3.** **Sensitivity analysis for robustness of SBS4-like mutational signature.** **(A)** Substitution profiles of the SBS4-like mutational signatures extracted in sensitivity analyses by fixing the non-negative matrix factorization rank k = 6, 7, and 8. **(B)** Cosine similarity among the reference COSMIC SBS4 mutational signature and the SBS4-like mutational signatures extracted in sensitivity analyses (k = 6, 7, and 8).


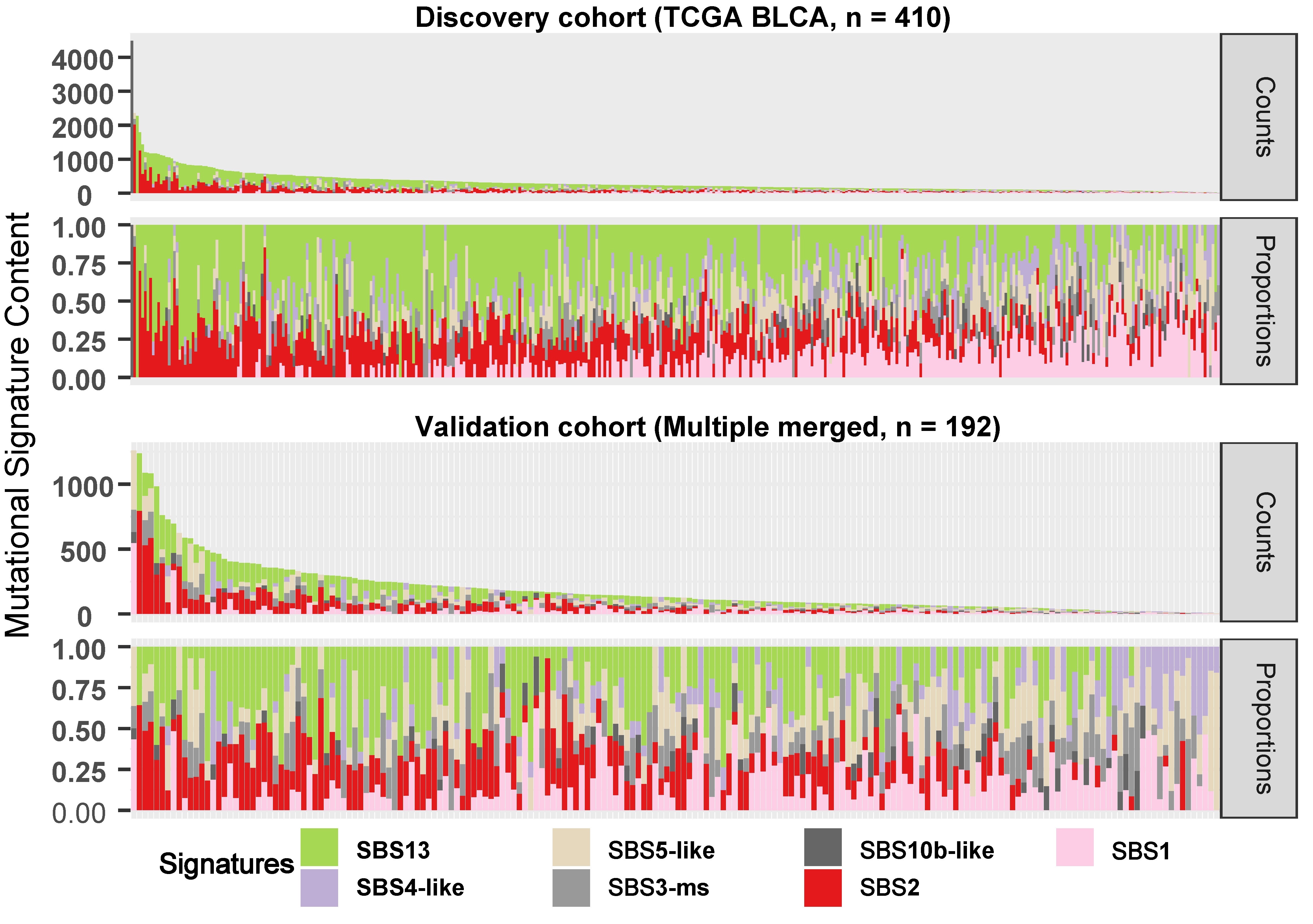


**Figure S4. Tumor-wise mutational signature exposure profiles are counted and proportioned in two independent cohorts.**


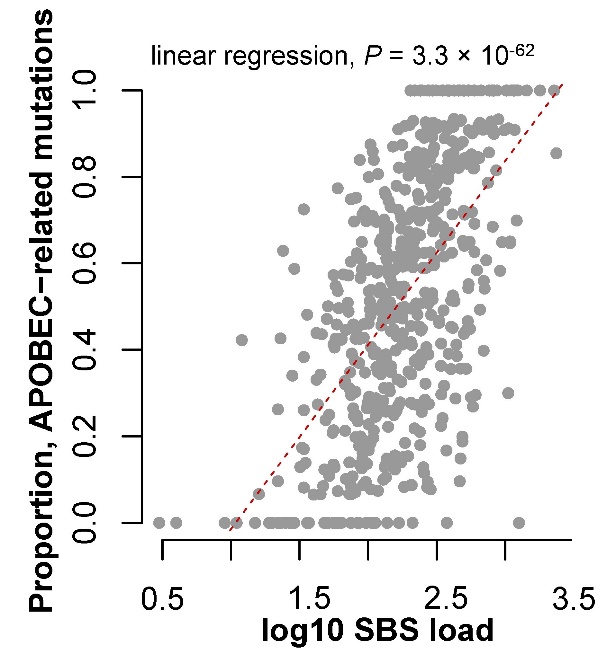


**Figure S5. Relationship between tumor mutation burden and proportion of APOBEC3-induced mutations. Each dot represents a tumor and the dashed red line demonstrates the linear regression line. *P*-value caculated by linear regression model.**

**Figure S6. Relationship between tumor mutation burden and proportion of SBS4-like mutations. Each dot represents a tumor and the dashed red line demonstrates the linear regression line. *P*-value caculated by linear regression model.**


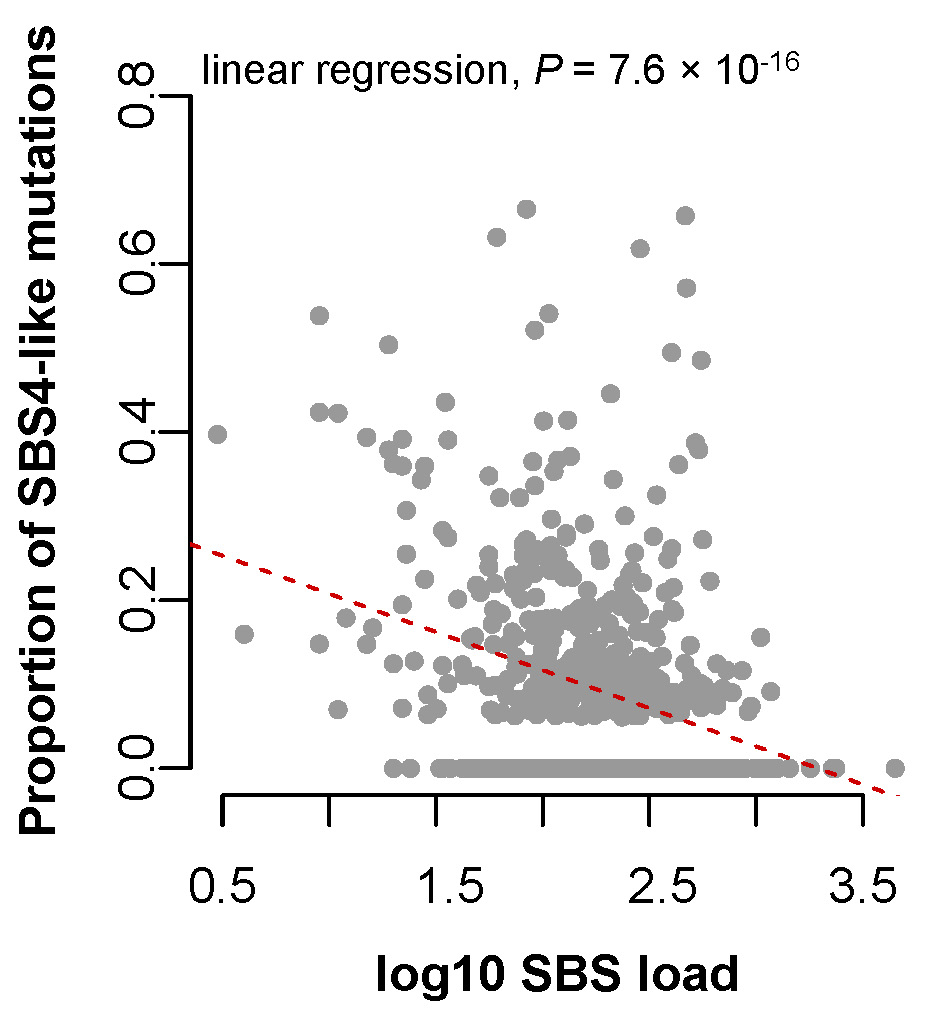

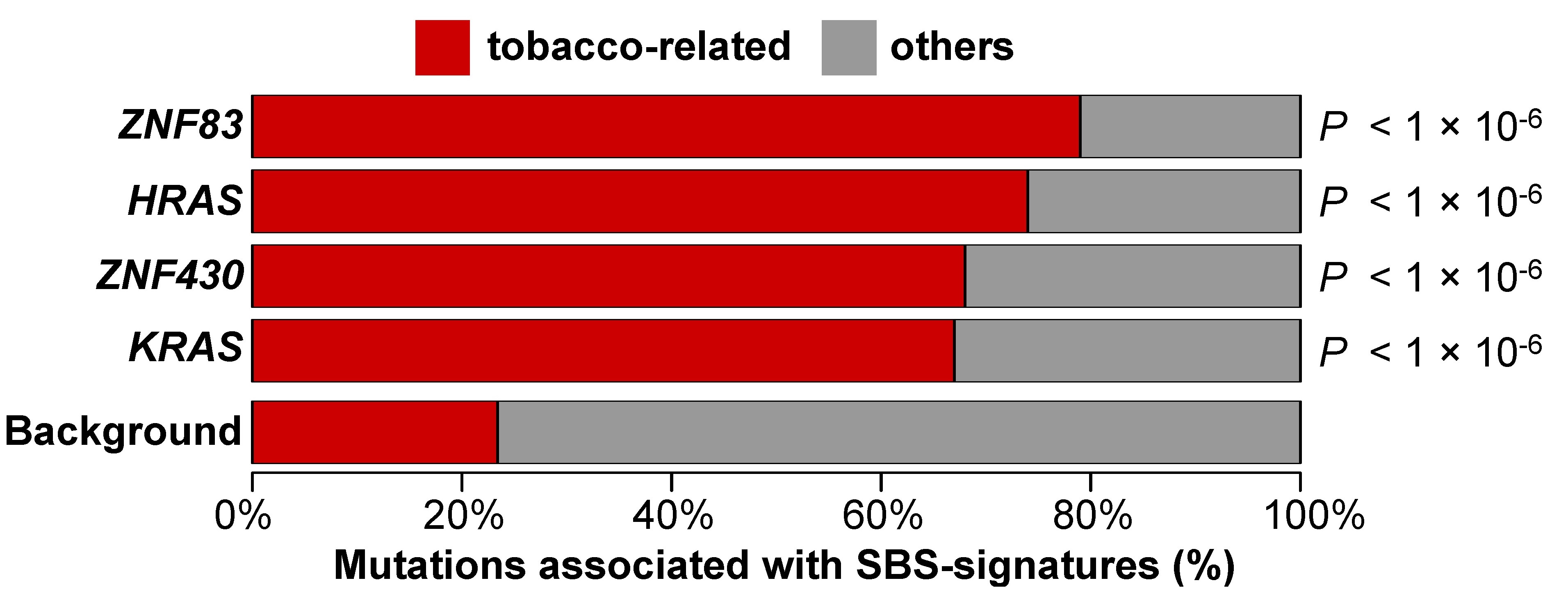


**Figure S7. Contribution of tobacco-related mutational signatures in mutations in BCa driver genes including *ZNF83*, *HRAS*, *ZNF430*, and *KRAS*.**

**Figure S8. No correlation between SBS4-like mutation load and patient sex.**


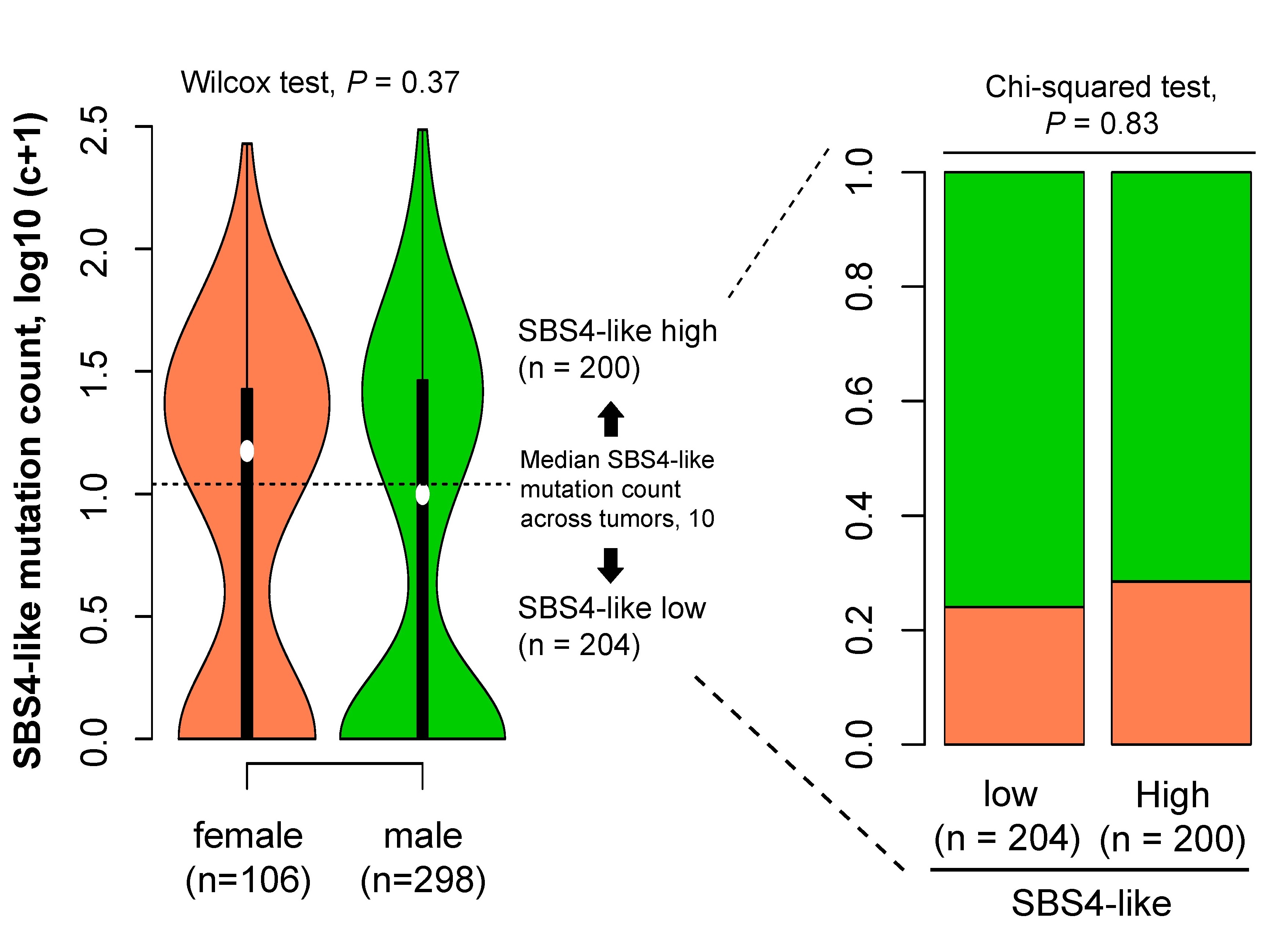

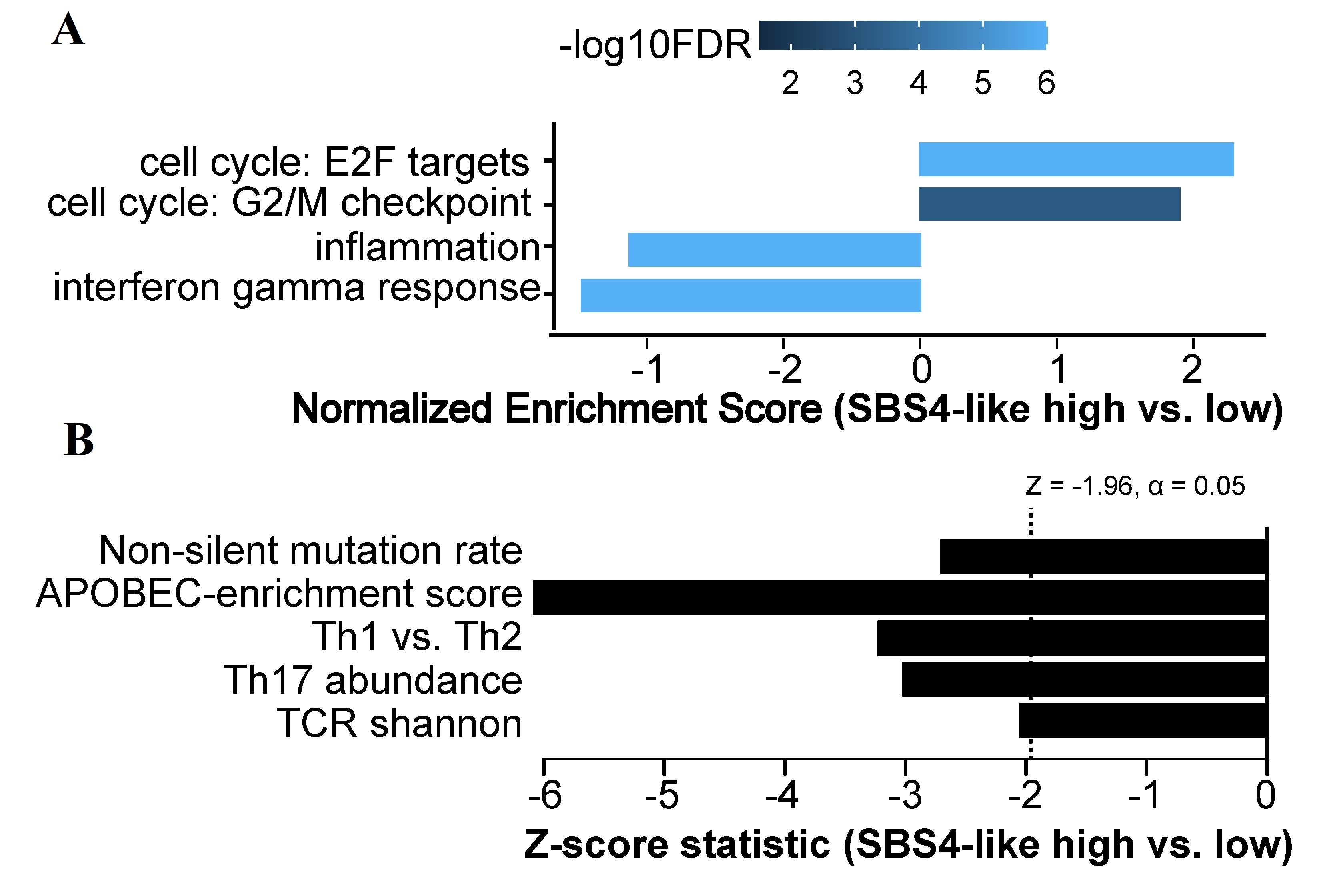


**Figure S9. (A) GSEA analysis of DEG (SBS4-like high versus SBS4-like low tumors).** Positive normalized enrichment scores indicate higher expression in SBS4-like high tumors and vice versa. **(B) The relationship between SBS4-like mutation load and non-silent mutation rate, APOBEC enrichment, and T-cell characteristics was assessed using the Wilcoxon test.** A negative Z-score indicates a lower level in SBS4-like high tumors. An absolute Z-score > 1.96 shows a statistically significant difference.


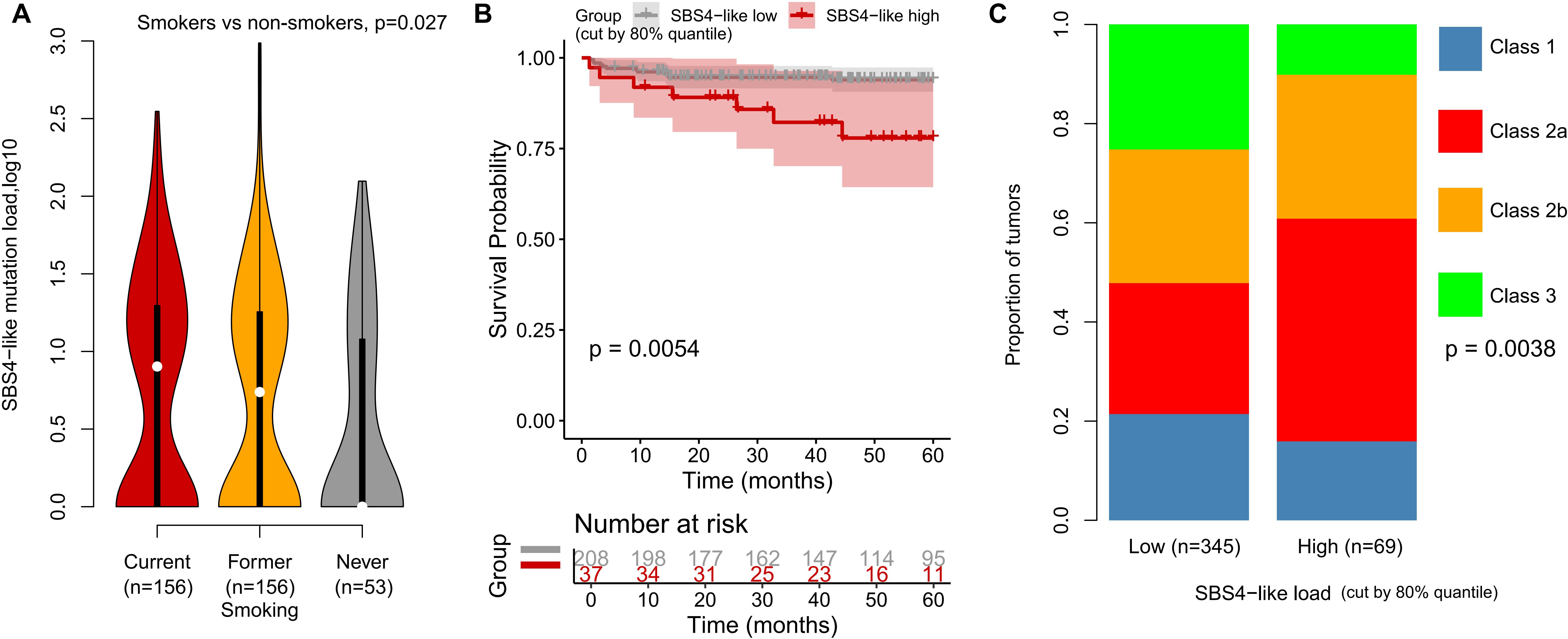


**Figure S10. Correlation between SBS4-like mutation load and smoking status (A), progression-free survival (B) and molecular subtypes (C) in UROMOL NMIBC tumors. (Data from Prip et al. Nature Genetics, 2025. PMID: 39753772.)**


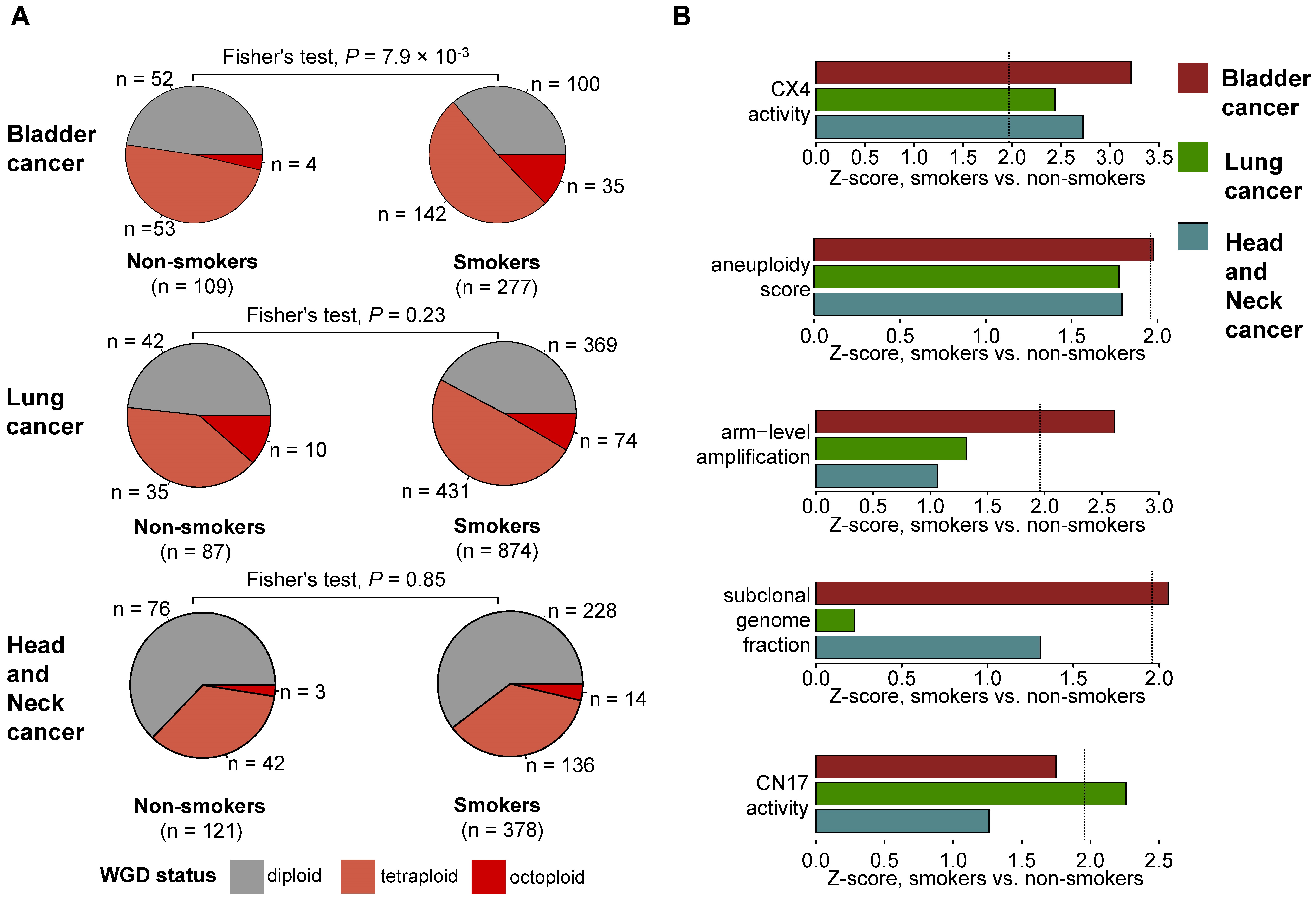


**Figure S11. Tobacco-smoking and tumor genomic instability. (A)** Relationship between tobacco smoking (never smokers versus ever smokers) and whole-genome duplication (WGD) in MIBC (upper), lung (middle), and head-and-neck cancers (lower). *Fisher’s exact test calculated P-values*. **(B)** Tobacco-related genomic instability features in MIBC and lung and head-and-neck cancers were analyzed using the Wilcoxon test. A positive Z-score indicates a high level of smokers as compared with non-smokers. The dashed line represents the threshold for statistical significance, where an absolute Z-score > 1.96 shows a statistically significant difference. CN17, copy number signature 17, related to homologous repair deficiency (HRD); CX3, chromosomal instability signature 3, associated with HRD and nucleotide-excision repair deficiency (NERD); CX4, chromosomal instability signature 4, associated with WGD.


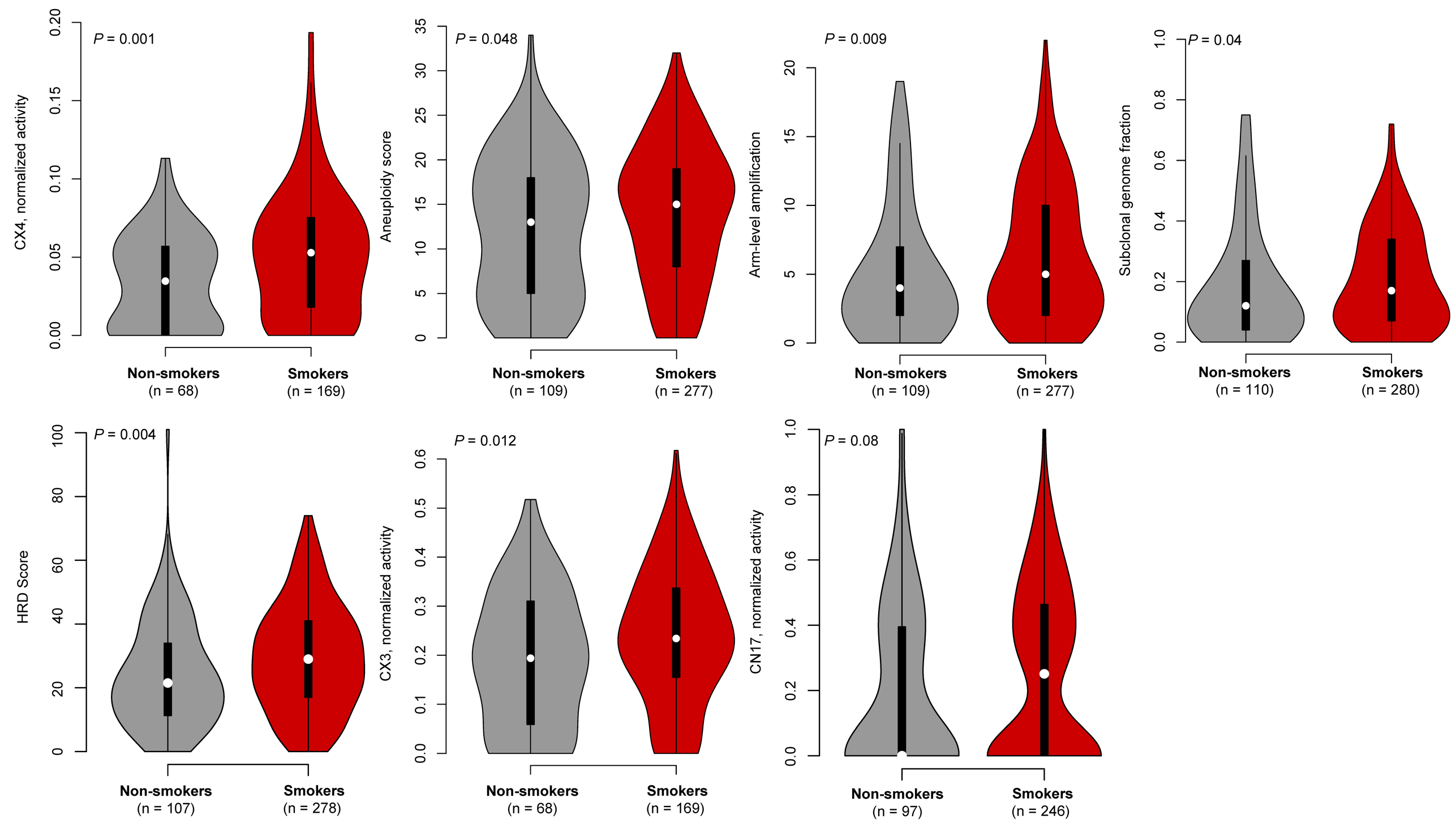


**Figure S12. Relationship between tobacco-smoking and genomic instability features in BCa.** Aneuploidy score was the sum of arm-level amplifications and deletions, which was higher in smokers. HRD score was the sum of the number of subchromosomal regions with allelic imbalance extending to the telomere, the number of chromosomal breaks between adjacent regions of at least 10Mb, and the number of LOH regions of intermediate size (> 15MB but < whole chromosome in length), which was higher in smokers. Chromosomal instability signature CX4 is characterized by a unique pattern of copy number change with neighboring segments separated by two copy changes and associated with genome duplication. Typical HRD features associated with impaired nucleotide excision repair and DNA damage sensing characterize chromosomal instability signature CX3. CN17, a copy number alteration signature associated with HRD. The Wilcoxon test calculated P-values.


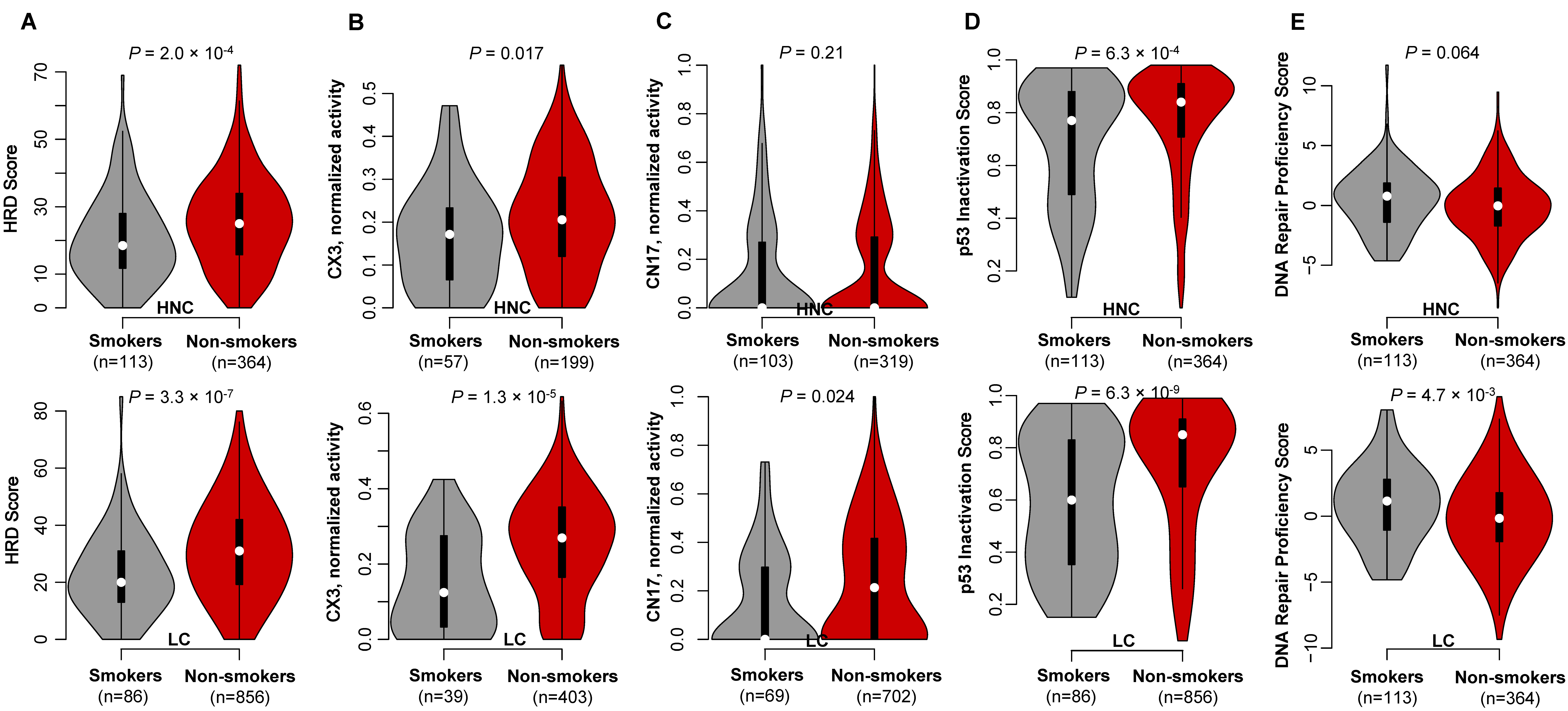


**Figure S13.** **Chromosomal instability and copy number signatures associated with smoking in TCGA lung and head-and-neck cancers. (A-B)** Significantly higher HRD score and higher activity of HRD/NERD-related chromosomal instability signature CX3 in smokers in both head-and-neck (upper) and lung cancer (lower) patients. **(C)** Poor presence of HRD-related copy number alteration signature CN17 in head-and-neck cancer (upper) and significantly higher CN17 activity in smokers in lung cancer patients (lower).


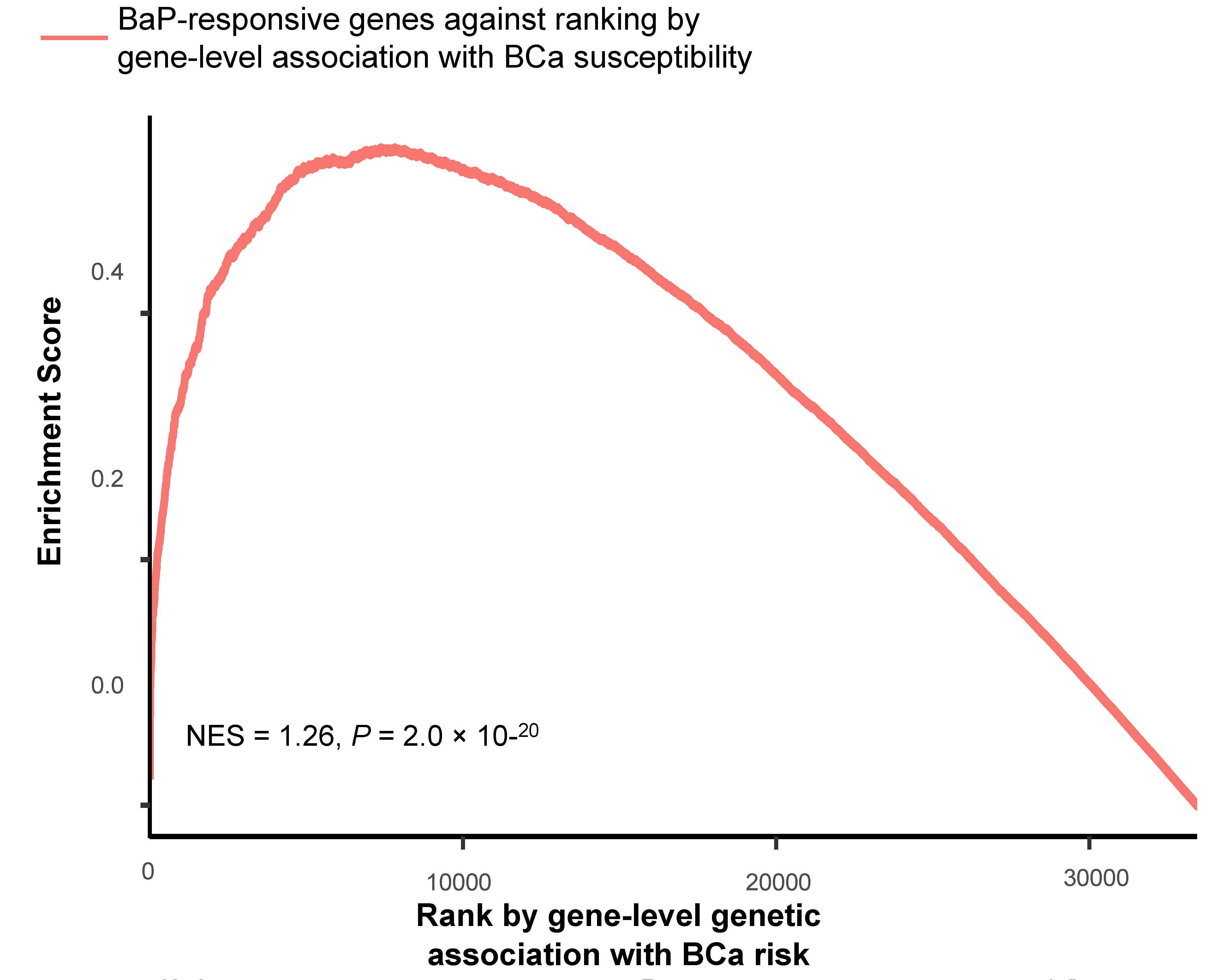


**Figure S14. Enrichment plot of GSEA analysis taking into account gene-level association with BCa risk and using DEG after BaP treatment in RT4 as the gene-set of interest.**

**Table S1. Overlap genes within the top 100 genetic association with BCa risk and significantly perturbed upon BaP treatment**

| **Gene** | **Description** | **Cytoband** | **Sig._BCa_^a)^** | **Sig._BaP_^b)^** |
| --- | --- | --- | --- | --- |
| *FGFR3* | Fibroblast Growth Factor Receptor 3 | 4p16.3 | 36.03 | 18.33 |
| *TACC3* | Transforming Acidic Coiled-Coil Containing Protein 3 | 4p16.3 | 39.99 | 13.72 |
| *SLBP* | Stem-Loop Binding Protein | 4p16.3 | 24.05 | 22.01 |
| *SULT1A2* | Sulfotransferase Family 1A Member 2 | 16p11.2 | 12.31 | 1.38 |
| *NUPR1* | Nuclear Protein 1, Transcriptional Regulator | 16p11.2 | 8.68 | 85.27 |
| *CLN3* | CLN3 Lysosomal/Endosomal Transmembrane Protein, Battenin | 16p12 | 7.49 | 13.70 |
| *APOBR* | Apolipoprotein B Receptor | 16p12 | 7.58 | 1.97 |
| *SIVA1* | SIVA1 Apoptosis Inducing Factor | 14q32.33 | 8.48 | 14.95 |
| *PSCA* | Prostate Stem Cell Antigen | 8q24.3 | 27.49 | 54.96 |
| *LYNX1* | Ly6/Neurotoxin 1 | 8q24.3 | 13.91 | 3.41 |
| *LY6D* | Lymphocyte Antigen 6 Family Member D | 8q24.3 | 14.13 | 25.20 |
| *LY6K* | Lymphocyte Antigen 6 Family Member K | 8q24.3 | 15.27 | 37.01 |
| *MICAL1* | Microtubule Associated Monooxygenase, Calponin And LIM Domain Containing 1 | 6q21 | 8.79 | 5.98 |
| *RPS11* | Ribosomal Protein S11 | 19q13.3 | 7.74 | 10.35 |
| *ATP1B1* | ATPase Na+/K+ Transporting Subunit Beta 1 | 1q24.2 | 8.66 | 13.73 |
| *NDUFB8* | NADH: Ubiquinone Oxidoreductase Subunit B8 | 10q24.31 | 7.75 | 7.86 |
| *TERT* | Telomerase Reverse Transcriptase | 5p15.33 | 12.09 | 7.41 |
| *PAG1* | Phosphoprotein Membrane Anchor With Glycosphingolipid Microdomains 1 | 8q21.13 | 12.89 | 3.98 |
| *FBN1* | Fibrillin 1 | 15q21.1 | 10.25 | 2.35 |
| *BANF1* | BAF Nuclear Assembly Factor 1 | 11q13.1 | 7.57 | 6.51 |
| *C1QTNF6* | C1q And TNF Related 6 | 22q12.3 | 9.55 | 27.73 |

^a)^Significance of gene-level genetic association with BCa risk, -log10 P-values; ^b)^Significance of differential expression between BaP treated and non-treated RT4 cells, -log10 adjusted P-values;

**Table S2. Known or possible driver genes (curated in Shi, Meng et al. Genome Medicine, 2020. PMID:** **32988402) perturbed by BaP treatment in RT4 cells.**

| **Gene** | **Gene type** | **log2FC** | **FDR** |
| --- | --- | --- | --- |
| *ZFP36L2* | Possible TSG | -0.89 | 1.24E-29 |
| *CDKN2C* | Possible TSG | -1.26 | 2.71E-22 |
| *RPL5* | Known TSG | -0.58 | 2.39E-14 |
| *PTMA* | Possible TSG | -0.80 | 3.19E-13 |
| *CCND1* | Known oncogene | 0.69 | 6.11E-13 |
| *RPL22* | Possible TSG | -0.59 | 4.70E-12 |
| *PSIP1* | Known TSG | -0.62 | 4.78E-10 |
| *RUNX1* | Known TSG | -0.43 | 1.62E-05 |
| *DAZAP1* | Possible TSG | -0.42 | 3.22E-05 |
| *CHD3* | Possible TSG | -0.35 | 1.05E-04 |
| *MYC* | Known oncogene | 0.47 | 5.45E-04 |
| *ATF7IP* | Known TSG | -0.31 | 1.55E-03 |
| *FUBP1* | Known TSG | -0.33 | 6.32E-03 |
| *MAPK1* | Known oncogene | 0.28 | 7.35E-03 |
| *IDH1* | Known oncogene | 0.28 | 9.35E-03 |
| *AXIN1* | Known TSG | -0.28 | 1.31E-02 |
| *GNA13* | Known oncogene | 0.19 | 2.94E-02 |
| *KRAS* | Known oncogene | 0.31 | 3.07E-02 |
| *GATA3* | Possible TSG | -0.27 | 3.18E-02 |
| *KMT2A* | Known TSG | -0.21 | 3.42E-02 |
| *PLXNB2* | Possible oncogene | 0.22 | 3.53E-02 |
| *ATM* | Known TSG | -0.27 | 3.64E-02 |
| *BRAF* | Known oncogene | 0.32 | 3.78E-02 |
| *SOS1* | Known oncogene | 0.23 | 3.98E-02 |

FC, fold change; FDR, false-discovery rate; TSG, tumor suppressor gene.
