# Supplementary material for: Tobacco's dual genomic footprints in bladder cancer revealed by multi-omics analysis: An SBS4-like LumU-enriched signature and smoking-driven HRD-related genomic instability
Source: Genes Dis. 2025 Dec 23;13(6):102001. doi: 10.1016/j.gendis.2025.102001 (PMC13380158; doi:10.1016/j.gendis.2025.102001)
Supplement: Multimedia component 2 [file mmc2.docx]

**SUPPLEMENTARY MATERIALS AND METHODS**

**Data used**

Whole-exome sequencing (WES) somatic substitution variant calls (n = 410; #mutations = 131,660), WES somatic copy-number variant (CNV) calls (n = 408), RNA-seq transcriptomes (n = 410, RSEM normalized counts), and patient clinical data for TCGA BCa were downloaded from the cBioPortal database (TCGA Cell 2017 data package) (1). WES data from a non-TCGA cohort of 192 BCa, previously used for our publication (2), were used to validate de novo extracted mutational signatures. COSMIC mutational signatures (version 3.2) in numeric format were obtained from the COSMIC Mutational Signature database (3). Raw RNA-seq counts of TCGA BCa tumors and genome-wide CpG methylomes of TCGA BCa tumors measured with the Illumina 450K array were obtained from the UCSC Xena data portal (4). Single-cell RNA-seq data of a BaSq MIBC tumor were downloaded from the GEO database (accession number GSE145137) (5). Markers for human skin basal and suprabasal keratinocytes were extracted from The Single Cell Type Atlas of The Human Protein Atlas (THPA) database (6). The GWAS meta-analysis summary statistics (study accession ID: GCST90011817) involved data from two large population-based cohorts of subjects of European ancestry (n = 412,592, including 2,242 BCa cases) and provided BCa genetic-association *P*-values for 9,987,520 variants (7).

**De novo Mutational Signature Extraction**

Non-negative matrix factorization (NMF)-based de novo mutational signature extraction was applied to the WES single base substitutions (SBS) calls of TCGA BCa tumors. The optimal number of signatures was automatically estimated using cophenetic correlation coefficients and residual sum of squares (RSS) as previously described (8). Sensitivity analysis was performed by fixing the number of signatures to 6 and 8 to test the robustness of the SBS4-like signature. De novo mutational signatures were compared against known COSMIC signatures (v3.2) by calculating cosine similarity as described (8). For further validation, de novo extracted mutational signatures in the 192 non-TCGA cohorts were deconvoluted, and the exposure of the mutational signature in these samples was estimated using the Palimpsest Bioconductor package v2.0 with default parameters (9).

**Transcriptional Strand Bias Analysis**

The distribution of mutated purine versus pyrimidine bases on the untranscribed versus transcribed strands of genes was analyzed. Odds ratios were calculated, and 1000-times permutations were performed for statistical significance testing using the background of all mutations as the reference.

**Mutational Origin Analysis.**

A probabilistic approach proposed by Letouzé and colleagues was applied to infer the mutagenic origin of a given mutation, represented by mutational signatures. The probability that a given mutational signature gave rise to a specific mutation event in a given tumor was calculated as described previously (8), using the Palimpsest Bioconductor package v2.0 with default parameters.

**Genomic Instability Features.**

Chromatin instability signatures were calculated as described in Drews et al (10). Copy number alteration signatures were calculated as described in Steele et al (11). Whole genome duplication, aneuploidy (number of arm-level copy number alterations), and subclonal genome fractions were calculated using the ABSOLUTE pipeline as described in Taylor et al (12). The HRD score, defined as the sum of the number of subchromosomal regions with allelic imbalance extending to the telomere, the number of chromosomal breaks between adjacent regions of at least 10Mb, and the number of loss of heterozygosity (LOH) regions of intermediate size (>15MB but <whole chromosome in length), was calculated as described previously (13).

**Transcriptome-Based Molecular Subtyping**

The consensus clustering classifiers by Kamoun et al. were applied to the log2-transformed RSEM transcriptome of TCGA BLCA tumors to classify the tumors into a total of 6 molecular subtypes (Luminal Papillary, LumP; Luminal Unstable, LumU; Luminal Non-specified, LumNS; Basal/Squamous, Ba/Sq; Stroma-rich; and Neuroendocrine-like, NE-like) (14). Samples unable to be confidently assigned to any molecular subtype were discarded.

**Transcriptomic Differential Analysis**

Raw RNA-seq counts of TCGA BLCA tumors were downloaded from UCSC Xena data portal and compared against the SBS4-like mutation burden group dichotomized at the median. The DESeq2 R package was used for transcriptome-wide differential analysis. Hypergeometric test-based enrichment analysis, as well as gene set enrichment analysis (GSEA) based on the pre-ranking by log2 fold-change (log2FC), were performed using the clusterProfiler R package.

**Tumor Microenvironment Analysis**

The constitution of the tumor stroma, including infiltrated immune and mesenchymal lineages, was computationally estimated using Microenvironment Cell Populations-counter (MCP-counter), based on the tumor transcriptomes, with default parameters (15). The relative abundance of T cells, CD8 T cells, NK cells, cytotoxic lymphocytes, B lineage, monocytic lineage, myeloid dendritic cells, neutrophils, endothelial cells, and fibroblasts was inferred. The neo-antigen load, TCR diversity, and abundance of Th1, Th2, and Th17 cells were estimated as described in Thorsson et al (16).

**APOBEC-Mutagenesis Analysis**

In addition to the de novo extracted APOBEC mutational signature, we also analyzed the APOBEC enrichment score and estimated minimal mutation load attributed to APOBEC as additional measurements of APOBEC-related mutation load in BCa tumors, using the P-MACD pipeline as described in Roberts et al (17).

**Single-Cell RNA-seq Analysis**

Log2 transcripts-per-million normalized gene expression of single cells from a BaSq subtype MIBC tumor was downloaded from GSE145137 dataset (5). Genes detected in fewer than 3 cells were discarded. Quality control excluded cells with fewer than 200 genes. Data were scaled, and the top 2000 variable genes were used as features for subsequent principal component analysis-based linear dimension reduction. The first 9 principal components were used for a graph-based clustering approach plus Louvain modularity optimization, and uniform manifold approximation and projection (UMAP) embedding for visualization. Differentially expressed genes of each cluster, i.e., cluster markers, were identified and used for annotation of the clusters. The highly expressed genes of the differentiated luminal tumor cells against the basal tumor cells were used as a signature of urothelial differentiation in the tumor, as previously described (18). All these analyses were performed using the Seurat v4 package with default parameters unless otherwise specified (19).

**Cell Culture**

The RT4 bladder transitional cell carcinoma cell line was purchased from Procell Life Science & Technology Co., Ltd. (Wuhan, China). The cell line was authenticated by the supplier using short tandem repeat (STR) profiling analysis. RT4 cells were cultured in McCoy’s 5A medium supplemented with 10% fetal bovine serum (FBS) and 1% penicillin-streptomycin under standard culture conditions suggested by the supplier. The cells were routinely checked for mycoplasma contamination.

**BaP Treatment and Bulk RNA-Sequencing**

We employed the RT4 cell line, derived from NMIBC and exhibits high xenobiotic-sensing AHR regulon activity (2), enabling metabolic activation of BaP into the DNA-reactive BPDE metabolite. MIBC cell lines, which typically exhibit low AHR activity and impaired BaP metabolic activation, would be less suited for capturing these initiating transcriptional effects. RT4 cells were seeded in triplicate in 96-well plates and left to adhere overnight before being treated with 5uM BaP or DMSO vehicle for 24h. The assays were done in three replicates for each treatment group. Total RNAs were extracted and subjected to quality control for purity, integrity, contamination, and concentration. Total RNA free of degradation, DNA, and protein contamination were processed for library preparation, which was then quantified with Qubit 2.0, assessed with Agilent 2000 for insert size, and then quantified with qPCR. The quality-controlled library was sequenced using 150bp pair end reads on the Illumina NovaSeq platform. The fastq files were trimmed for adapter sequences and quality, and then mapped to the human reference genome hg19 using the STAR package v2.5.4b. Raw read counts were then calculated using the subread featureCounts v1.6.0 package. The BaP perturbation signature genes were defined as genes with an absolute log2 fold-change > 0.58 and false discovery rate-adjusted P-value < 0.05 in DESeq2 differential expression analysis.

**Gene-Level Genetic Association with BCa Risk**

Variant-to-gene mapping was applied to the GWAS summary statistics using five independent approaches, including the versatile gene-based association study (VEGAS), meta-analysis gene-set enrichment of variant associations (MAGENTA), multi-marker analysis of genomic annotation (MAGMA), functional summary-based imputation (FUSION), and transcriptome-wide Mendelian randomization (TWMR) (20-23). The latter two involved the integration of GWAS and eQTL signals. For the FUSION analysis, the cross-tissue weights for cross-tissue features generated through sparse canonical correlation analysis on GTEx gene expression version 8 was used (24). For the TWMR analysis, the cross-tissue eQTL relationships were used as the exposure statistics, and those with a *P*-value < 5 × 10^-8^ were considered instruments for two-sample MR analyses using the inverse-variance weighted model. With each tool, a P-value was calculated for each gene regarding its association with BCa. The P-values of each individual approach were pooled by combination using Stouffer’s method. The genes were then ranked based on the –log10 transformation of the pooled P-values, with a higher score suggesting a higher likelihood of implication in BCa susceptibility.

**Statistical Analysis**

Unless otherwise specified, continuous variables were described as median with inter-quartile range (IQR) and compared using the Wilcoxon rank-sum test, and categorical variables as count with proportion were compared using the chi-squared test. Survival analysis was performed with Kaplan-Meir curves, log-rank test, and Cox proportional hazard modeling. All statistical analyses were conducted using R version 4.1.2. A two-sided P-value ≤ 0.05 was considered statistically significant.

**REFERENCES**

1. Gao J, Aksoy BA, Dogrusoz U, Dresdner G, Gross B, Sumer SO, et al. Integrative analysis of complex cancer genomics and clinical profiles using the cBioPortal. Science signaling. 2013;6(269):pl1.

2. Shi MJ, Meng XY, Fontugne J, Chen CL, Radvanyi F, Bernard-Pierrot I. Identification of new driver and passenger mutations within APOBEC-induced hotspot mutations in bladder cancer. Genome Med. 2020;12(1):85.

3. Tate JG, Bamford S, Jubb HC, Sondka Z, Beare DM, Bindal N, et al. COSMIC: the Catalogue Of Somatic Mutations In Cancer. Nucleic acids research. 2019;47(D1):D941-D7.

4. Goldman MJ, Craft B, Hastie M, Repecka K, McDade F, Kamath A, et al. Visualizing and interpreting cancer genomics data via the Xena platform. Nature biotechnology. 2020;38(6):675-8.

5. Lee HW, Chung W, Lee HO, Jeong DE, Jo A, Lim JE, et al. Single-cell RNA sequencing reveals the tumor microenvironment and facilitates strategic choices to circumvent treatment failure in a chemorefractory bladder cancer patient. Genome medicine. 2020;12(1):47.

6. Karlsson M, Zhang C, Mear L, Zhong W, Digre A, Katona B, et al. A single-cell type transcriptomics map of human tissues. Sci Adv. 2021;7(31).

7. Rashkin SR, Graff RE, Kachuri L, Thai KK, Alexeeff SE, Blatchins MA, et al. Pan-cancer study detects genetic risk variants and shared genetic basis in two large cohorts. Nature communications. 2020;11(1):4423.

8. Letouze E, Shinde J, Renault V, Couchy G, Blanc JF, Tubacher E, et al. Mutational signatures reveal the dynamic interplay of risk factors and cellular processes during liver tumorigenesis. Nature communications. 2017;8(1):1315.

9. Shinde J, Bayard Q, Imbeaud S, Hirsch TZ, Liu F, Renault V, et al. Palimpsest: an R package for studying mutational and structural variant signatures along clonal evolution in cancer. Bioinformatics. 2018;34(19):3380-1.

10. Drews RM, Hernando B, Tarabichi M, Haase K, Lesluyes T, Smith PS, et al. A pan-cancer compendium of chromosomal instability. Nature. 2022;606(7916):976-83.

11. Steele CD, Abbasi A, Islam SMA, Bowes AL, Khandekar A, Haase K, et al. Signatures of copy number alterations in human cancer. Nature. 2022;606(7916):984-91.

12. Taylor AM, Shih J, Ha G, Gao GF, Zhang X, Berger AC, et al. Genomic and Functional Approaches to Understanding Cancer Aneuploidy. Cancer Cell. 2018;33(4):676-89 e3.

13. Marquard AM, Eklund AC, Joshi T, Krzystanek M, Favero F, Wang ZC, et al. Pan-cancer analysis of genomic scar signatures associated with homologous recombination deficiency suggests novel indications for existing cancer drugs. Biomark Res. 2015;3:9.

14. Kamoun A, de Reynies A, Allory Y, Sjodahl G, Robertson AG, Seiler R, et al. A Consensus Molecular Classification of Muscle-invasive Bladder Cancer. European urology. 2020;77(4):420-33.

15. Becht E, Giraldo NA, Lacroix L, Buttard B, Elarouci N, Petitprez F, et al. Estimating the population abundance of tissue-infiltrating immune and stromal cell populations using gene expression. Genome biology. 2016;17(1):218.

16. Thorsson V, Gibbs DL, Brown SD, Wolf D, Bortone DS, Ou Yang TH, et al. The Immune Landscape of Cancer. Immunity. 2018;48(4):812-30 e14.

17. Robertson AG, Kim J, Al-Ahmadie H, Bellmunt J, Guo G, Cherniack AD, et al. Comprehensive Molecular Characterization of Muscle-Invasive Bladder Cancer. Cell. 2017;171(3):540-56 e25.

18. Neyret-Kahn H, Fontugne J, Meng XY, Groeneveld CS, Cabel L, Ye T, et al. Epigenomic mapping identifies an enhancer repertoire that regulates cell identity in bladder cancer through distinct transcription factor networks. Oncogene. 2023;42(19):1524-42.

19. Hao Y, Hao S, Andersen-Nissen E, Mauck WM, 3rd, Zheng S, Butler A, et al. Integrated analysis of multimodal single-cell data. Cell. 2021;184(13):3573-87 e29.

20. Liu JZ, McRae AF, Nyholt DR, Medland SE, Wray NR, Brown KM, et al. A versatile gene-based test for genome-wide association studies. Am J Hum Genet. 2010;87(1):139-45.

21. Segre AV, Consortium D, investigators M, Groop L, Mootha VK, Daly MJ, et al. Common inherited variation in mitochondrial genes is not enriched for associations with type 2 diabetes or related glycemic traits. PLoS Genet. 2010;6(8).

22. de Leeuw CA, Mooij JM, Heskes T, Posthuma D. MAGMA: generalized gene-set analysis of GWAS data. PLoS Comput Biol. 2015;11(4):e1004219.

23. Gusev A, Ko A, Shi H, Bhatia G, Chung W, Penninx BW, et al. Integrative approaches for large-scale transcriptome-wide association studies. Nature genetics. 2016;48(3):245-52.

24. Feng H, Mancuso N, Gusev A, Majumdar A, Major M, Pasaniuc B, et al. Leveraging expression from multiple tissues using sparse canonical correlation analysis and aggregate tests improves the power of transcriptome-wide association studies. PLoS Genet. 2021;17(4):e1008973.
